# Submucosal lipoma of the sigmoid colon as a rare cause of mucoid diarrhea: a case report

**DOI:** 10.1186/s13256-016-0798-6

**Published:** 2016-01-20

**Authors:** S. U. B. Dassanayake, N. P. Dinamithra, N. M. M. Nawarathne

**Affiliations:** Gastroenterology & Hepatology Unit, National Hospital of Sri Lanka, Colombo, Sri Lanka

**Keywords:** Benign colonic tumors, Colonic lipoma, Endoscopic resection

## Abstract

**Background:**

Symptomatic presentations of colonic lipomas are very rare in clinical practice, and may mimic colonic malignancy. The likelihood of presenting symptoms has been shown to depend on the size of the lesion.

**Case presentation:**

We describe the case of a 72-year-old Sinhalese man presenting with worsening mucoid diarrhea who was subsequently diagnosed to have a lipoma of the sigmoid colon. His disease was successfully managed with endoscopic resection.

**Conclusion:**

Confidently establishing the rare diagnosis of a colonic lipoma usually requires a combination of endoscopic, radiological, and histological evaluation, and is therefore very challenging. With the advancement of endoscopic procedures, endoscopic resection is widely practiced as the definitive management of these cases.

## Background

Colonic lipomas are rare mesenchymal tumors that are usually asymptomatic. However, large lesions have been described to cause symptoms like abdominal pain, constipation, diarrhea, and even obstruction due to intussusception [1]. The epidemiological, macroscopic, and clinical presentation can sometimes suggest a malignant nature, although malignant transformation does not occur [2]. Their rarity and multitude of clinical presentations make colonic lipomas an underemphasized and misdiagnosed pathology [3].

## Case presentation

A 72-year-old Sri Lankan Sinhalese man presented to our clinic with a 3-month history of worsening mucoid diarrhea. Initially his diarrhea was infrequent and mild, but had gradually worsened to 15–20 episodes per day with associated tenesmus and occasional blood staining, significantly limiting his daily activity. He had no loss of appetite or weight loss. His medical history was unremarkable except for dyslipidemia, for which he was taking atorvastatin (10 mg daily). He had no history of past surgery. Results of an abdominal examination were normal and a digital rectal examination did not reveal any masses or blood. Results of laboratory investigations, including a full blood count, basic biochemistry, and tests for inflammatory markers, were within normal limits.

**Fig. 1:**
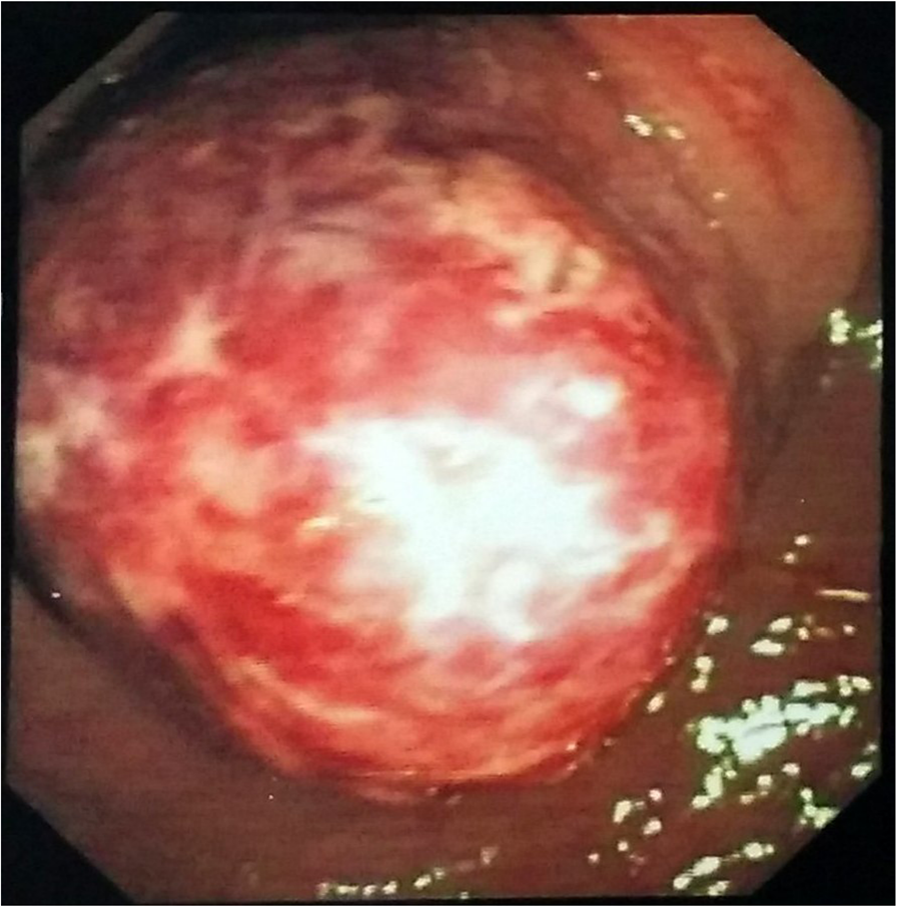
Initial endoscopic appearance of the lipoma

**Fig. 2:**
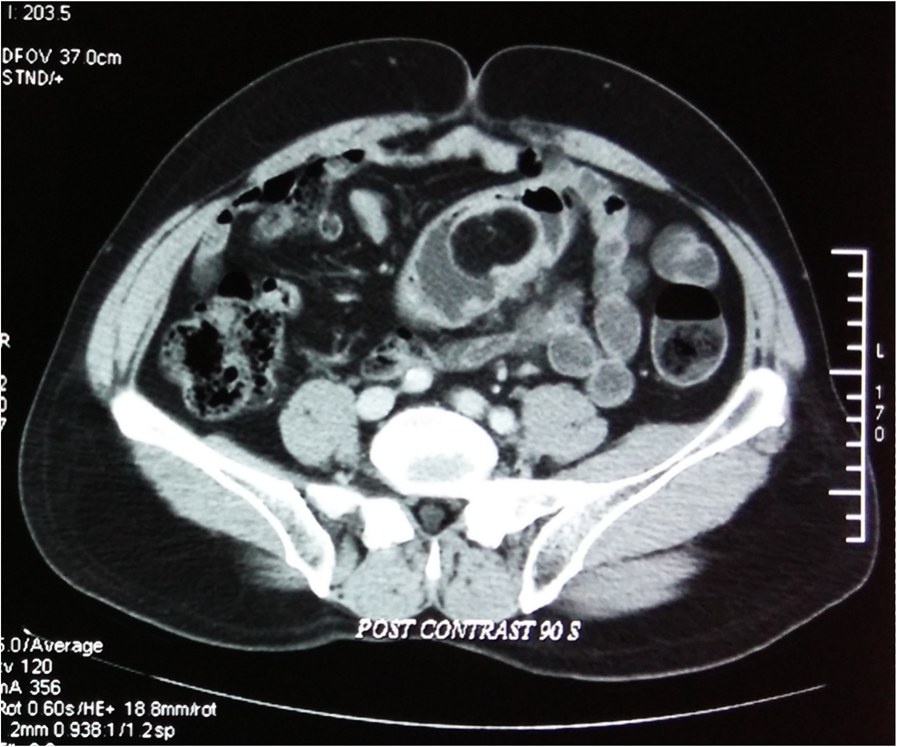
Contrast-enhanced computed tomography scan showing a hypodense lesion in the sigmoid colon

Subsequently, our patient had a colonoscopy, which revealed a single 3 × 2 cm pedunculated, spherical, smooth mass with minimal surface ulceration in his lower sigmoid colon (Fig. 1). Initial biopsies revealed only inflammatory tissues. A triple-contrast computed tomography scan of his abdomen revealed a 2.8 × 2.2 cm rounded mass in his sigmoid colon causing mild narrowing of the lumen (Fig. 2). The density was compatible with that of fatty tissue. A repeat endoscopy was performed and the lesion excised after applying a nylon wire loop to the stalk. The bulging cut surface was composed of yellow adipose tissue.

The procedure was uncomplicated and resulted in a dramatic improvement in our patient’s symptoms. A histological examination confirmed the presence of mature adipose tissue in a submucosal location. Sigmoidoscopy 2 weeks after the procedure showed a well-healed excision site.

## Discussion

Although colonic lipomas are the second most common benign colonic tumor, they are surprisingly rare. Since the first case was reported by Bauer in 1757, only about 300 cases have been recorded according to the literature [4]. A recent report by Rogy *et al* *.* estimates that colonic lipomas constitute about 0.3 % of cases involving colorectal problems and 1.8 % of cases of benign colorectal disease [1]. More than 75 % of lesions occur in the right colon, and about 10 % are multiple, especially in the region of the cecum [5]. Ninety percent of colonic lipomas are submucosal in origin, the rest are located in the subserosal or intramucosal layers. There is a slight female preponderance according to some reports, with left-sided tumors being more common in males patients [2].

Lipomas are usually found incidentally during imaging or colonic surgery for other indications. The probability of being symptomatic correlates with the size of the lipoma; lesions larger than 4 cm become symptomatic in 75 % of cases [2]. The symptoms can vary with the location and the size of the tumor, and include abdominal pain, change in bowel habits, rectal bleeding, obstruction, perforation, intussusception, prolapse, and even massive hemorrhage. Large lesions can develop surface ulceration that leads to bleeding, giving rise to a varied combination of symptoms.

Radiographical investigations like barium enema, computed tomography (as in our case), or magnetic resonance imaging may assist in the diagnosis. However, lesions smaller than 2 cm are difficult to visualize radiologically and cases with complications like intussusception or bowel wall thickening may be misdiagnosed as malignancy [6]. Endoscopic ultrasound usually demonstrates a hyperechoic lesion localized to the submucosal layer, which would be diagnostic for lipoma [2]. Heterogeneous or hypoechoic lesions on endoscopic ultrasound have rarely been described.

Colonoscopy usually demonstrates a pedunculated or sessile smooth lesion that may demonstrate the “pillow” or “cushion” sign when indented with closed biopsy forceps—that is, it promptly reassumes its previous shape on release [6]. Superficial biopsies usually do not assist in the diagnosis owing to the submucosal location of the tumor.

Small (<2 cm) asymptomatic lesions, if unequivocally diagnosed, can be simply followed up as there is no significant malignant degeneration [2]. Larger lesions that are symptomatic require endoscopic or surgical removal. Adipose tissue has little water content to conduct electricity during endoscopic snare removal, requiring the endoscopist to use a larger electrical current, increasing the risk of thermal injury to the colonic wall and perforation. However, recent advances in endoscopic equipment and accessories have made the excision of pedunculated lesions safe and excision is now recommended [7]. Staged partial resections can be carried out if complete resection is not practical. Surgical resection is necessary in sessile lesions, lesions with extensions of the serosa or muscularis propria into the pedicle, or in cases complicated with bowel obstruction or intussusception [2].

## Conclusion

Establishing the rare diagnosis of a symptomatic colonic lipoma can prove challenging, requiring multidisciplinary input. However, once the tumor is confidently diagnosed, modern endoscopic techniques have revolutionized the management, making surgery necessary only in complicated cases.

## Consent

Written informed consent was obtained from the patient for publication of this case report and accompanying images. A copy of the written consent is available for review by the Editor-in-Chief of this journal.
